# Some small sample properties of the EWOC design in dose escalation trials

**DOI:** 10.1186/1745-6215-16-S2-P221

**Published:** 2015-11-16

**Authors:** Nelson Kinnersley

**Affiliations:** 1 Roche Products Ltd, Welwyn, UK

## 

Model-based approaches to dose escalation trials such as CRM, EWOC and their variants are becoming increasingly popular in Phase I oncology trials. Although the literature includes a number of reasons why the practitioner may want to consider using a model-based design over a more traditional 3+3 design, there are still a number of reasons why they are not universally adopted. Theoretical reasons have often been provided for their superiority over 3+3 designs and these have generally relied on large sample theory, whereas Phase I trial sizes are usually considerably lower. Furthermore, when information external to the planned trial is available and may be used to construct informative prior distributions, the small sample properties of model-based designs are less well understood.

By considering a planned trial for which only a discrete set of doses are available, we use simulation to show some small sample properties of the EWOC design (Babb et al, 1998). When the model recommends a dose between the available doses, we evaluate the strategy of always choosing the dose below that recommended by the model versus the closest available dose (even if that is higher). We also characterise two different parameterisations of the Bayesian 2-parameter logistic model that underpins the EWOC design and make some recommendations that could increase the acceptability of the EWOC design to those more comfortable with implementing a 3+3 design.

